# A deep learning drug screening framework for integrating local-global characteristics: A novel attempt for limited data

**DOI:** 10.1016/j.heliyon.2024.e34244

**Published:** 2024-07-14

**Authors:** Ying Wang, Yangguang Su, Kairui Zhao, Diwei Huo, Zhenshun Du, Zhiju Wang, Hongbo Xie, Lei Liu, Qing Jin, Xuekun Ren, Xiujie Chen, Denan Zhang

**Affiliations:** a Department of Pharmacogenomics, College of Bioinformatics Science and Technology, Harbin Medical University, Harbin, Heilongjiang, 150081, China; b The Fourth Hospital of Harbin Medical University, No.37 Yiyuan Street, Harbin, Heilongjiang, 150001, China; c College of Mathematics of Harbin Institute of Technology, No.92 Xidazhi Street, Harbin, Heilongjiang, 150001, China

**Keywords:** COVID-19, Virtual drug screening, Deep learning, Host-targeted antiviral drugs, Direct-acting antiviral drugs, Molecular docking

## Abstract

At the beginning of the "Disease X" outbreak, drug discovery and development are often challenged by insufficient and unbalanced data. To address this problem and maximize the information value of limited data, we propose a drug screening model, LGCNN, based on convolutional neural network (CNN), which enables rapid drug screening by integrating features of drug molecular structures and drug-target interactions at both local and global (LG) levels. Experimental results show that LGCNN exhibits better performance compared to other state-of-the-art classification methods under limited data. In addition, LGCNN was applied to anti-SARS-CoV-2 drug screening to realize therapeutic drug mining against COVID-19. LGCNN transcends the limitations of traditional models for predicting interactions between single drug targets and shows new advantages in predicting multi-target drug-target interactions. Notably, the cross-coronavirus generalizability of the model is also implied by the analysis of targets, drugs, and mechanisms in the prediction results. In conclusion, LGCNN provides new ideas and methods for rapid drug screening in emergency situations where data are scarce.

## Highlights

• Local and global features of drugs and targets are applied in deep learning models.
• Model has the potential for rapid drug screening for coronavirus infectious diseases.
• Predicted compounds may work through the anti-coronavirus universal pathway.

## Graphical abstract

**Figure:**
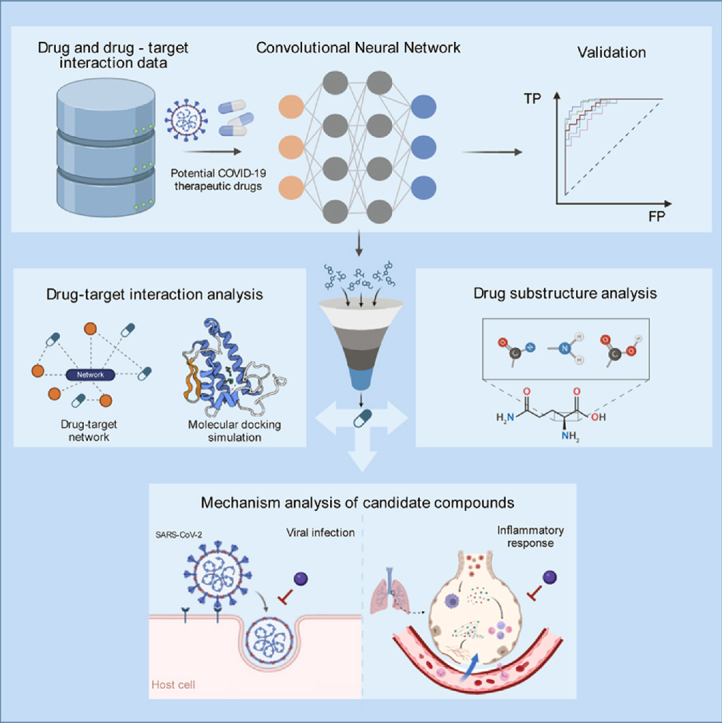

## 1 Introduction

Since the emergence of Severe Acute Respiratory Syndrome (SARS), Middle East respiratory syndrome (MERS), and COVID-19, there have been three global epidemic emergencies involving respiratory infectious diseases in the 21st century. SARS-CoV-2, similar to SARS-CoV and MERS-CoV, is an enveloped positive-strand RNA virus that belongs to the β-coronavirus genus of the coronavirus family. It is the seventh known coronavirus to infect humans [1,2]. The human-to-human transmission of SARS-CoV-2 has resulted in over 774 million confirmed cases of COVID-19 [3], and about 7 million deaths globally as of February 2024 (https://covid19.who.int/), leading to significant economic losses. This represents the most severe global health crisis since the 1918 influenza pandemic. Although the epidemic is approaching its end, the risk of future large-scale outbreaks of respiratory infectious diseases remains. WHO Director-General Tedros Adhanom Ghebreyesus has publicly warned of the possibility of an outbreak of "Disease X", arguing that a currently unknown pathogen causing human disease could lead to the next pandemic outbreak and calling for preparedness to deal with it. Therefore, it is imperative to develop a rapid drug screening method based on limited data for "Disease X" outbreaks.

Advancements in technology and the availability of diverse drug-related and disease-related data have facilitated the use of computational methods, particularly deep learning (DL) based virtual screening, in drug discovery, surpassing the efficacy of traditional computational methods [4]. Based on the combination of recurrent neural network and molecular docking technology, Joshi et al. [5] carried out virtual screening of potential SARS-CoV-2 main protease (Mpro) inhibitors in natural molecules and identified two anti-SARS-CoV-2 natural compounds, Palmatine and Sauchinone. Both of them showed the stability of binding to Mpro and the antagonistic ability against Mpro. Zhang et al. [6] used the 3CL protease of SARS-CoV-2 as a potential target, constructed a structural model based on its sequence features, and then used the deep full-sequence convolutional neural network (DFCNN) to learn the known binding and non-binding 3CL protease-ligand interaction data. Large-scale drug screening in several compound databases using trained models to identify reliable 3CL protease-ligand interactions. Das et al. [7] employed a message-passing neural network model based on geometric deep learning to extract structural information about drugs known to interact with ACE2 proteins to infer drugs that could be used in an experimental therapeutic regimen against COVID-19. All these exploration results have guiding significance for future anti-SRAS-CoV-2 small molecule experimental research, but none of them are free from the limitation that target features can only be based on a single known antiviral target.

Computational techniques employed in COVID-19 drug screening predominantly fall into five categories: sequence-based computational techniques, gene expression-based computational techniques, structure-based computational techniques, interaction-based computational techniques, and integrated approaches [8]. Sequence-based methods solely examine molecular and energy levels, overlooking the connection between viral infection and host targets. Gene expression-based methods achieve drug screening through signature matching [9] and neural network methods [10], but this method is considered to be very dependent on the perturbation of gene expression produced by drugs or diseases [9]. Structure-based methods generally use neural networks [11] or molecular docking [12] for drug screening, but most studies focus on a single target, and molecular docking depends largely on the cocrystal structure of the target protein. The interaction-based approach pays more attention to the interaction between viruses, targets, and drugs [13,14], but it also suffers from limitations due to the lack of specific feature information.

**Fig. 1:**
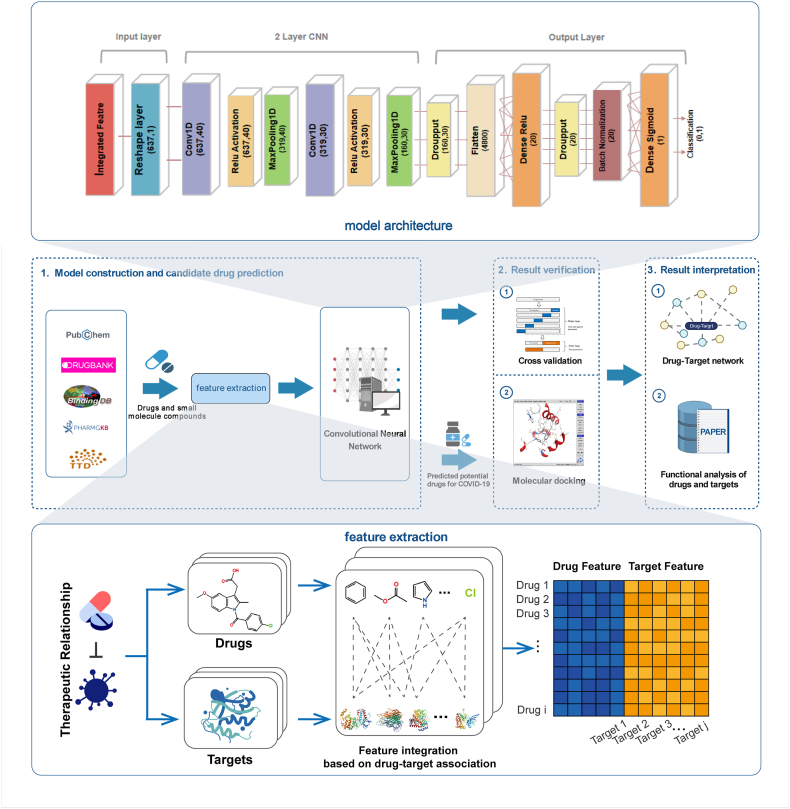
The overall workflow of the study. The combination of drug substructure features and drug-target association features is embedded as comprehensive feature vectors, which is trained by a convolutional neural network composed of double convolutional architecture. The performance of LGCNN and the accuracy of the predicted results are evaluated by multiple cross-validation and molecular docking simulation experiments. Drug-target network analysis and therapeutic mechanism analysis are used to explain the reliability of the predicted results.

Factors such as small sample size and unbalanced distribution of features in drug prediction have been a major challenge in computational prediction. An effective drug screening model should be capable of extracting sufficient feature information from the existing limited clinical drug data. We suggest integrating the representation of local-global therapeutic features, going beyond the traditional limitations of conducting studies on the three-dimensional structure and binding sites of a single target, and reducing the adverse effects of small sample sizes and distributional imbalances by enriching the feature information. This study proposes a deep-learning integrated model, namely LGCNN, to learn cross-drug substructures and their corresponding target features based on potential anti-SARS-CoV-2 drug sensitivity data from multiple authentic drug clinical trials (Fig. 1). Mapping between drug substructural features and targets constitute local structural features, while drug-target interaction features based on therapeutic effects reflect the global correlation between drugs, viruses, and hosts.

LGCNN is a deep learning model based on CNN, which can extract comprehensive compound information with potential therapeutic effects on COVID-19 from limited clinical data by fusing local and global therapeutic features, and screen out a list of candidate compounds from hundreds of thousands of potential compounds. Our results show that LGCNN outperforms multiple machine learning and neural network approaches in terms of performance, and molecular docking simulations and drug-target network analyses confirm the reliability of the prediction results based on the compound database. Notably, the high scores predicted compounds with the potential to exert therapeutic effects through multiple pathways. These pathways, which are shared across multiple coronavirus infections, exemplify the model's ability to generalize and provide a new approach for rapid screening in the face of unknown coronavirus outbreaks in the future. In addition, LGCNN integrates global features based on therapeutic mapping between drugs and multiple targets in the feature extraction process, and constructs local mappings of substructure features and target features, making it possible to predict multi-target drugs. In conclusion, this study proposes a drug screening model that integrates local-global therapeutic features, providing new ideas for establishing a rapid and efficient anticoronavirus drug screening platform to combat infectious disease pandemics.

## 2 Materials and methods

### 2.1 Dataset

Compound information for SARS-CoV-2 clinical trials was gathered from various databases including Pubchem [15], Drugbank [16], BindingDB [17], and TTD [18]. Integrate the SMILES [19] sequence information and drug-target associations of the compounds. The drug and target information related to potential COVID-19 treatments were processed to create drug-target corresponding structures. Subsequently, compounds and targets were further screened based on the species limitations (human and virus) of the targets. This yielded a total of 223 potential COVID-19 therapeutic drugs and 1036 drug-target correspondences (Table S1).

Candidate drug structures and targets data for prediction were obtained from databases including Drugbank [16], TTD [18], pharmGKB [20], PubChem [15], DGIdb [21], BioGRID [22], and BindingDB [17]. Drugs and small molecule compounds with SMILES structure and target information were extracted. The compounds were then screened further by restricting the source species and scope of the targets. A total of 210696 drugs and small molecules were obtained, of which 301785 drug-target interactions were obtained (Table S2).

### 2.2 Integration of local and global characteristics

LGCNN enables the discovery of multi-target therapeutics as well as therapeutics with novel holistic structures by identifying mapping associations between local drug substructures and targets under conditions of global relevance of therapeutic effects. Specifically, we use byte pair coding to extract structural information enriched in the SMILE sequence of the ligand and its therapeutic target information and combine the two to form a comprehensive feature vector.

The molecular access system fingerprints (MaccsKeys) [23] in the Python programming package RDKit were used to extract drug substructure feature vectors from the SMILES sequence. The substructure feature vectors of all compounds form the drug substructure matrix S. If the drug d(i) has a substructure s(j), then S(d(i),s(j)) is set to 1, otherwise 0. We constructed a target feature vector based on the therapeutic association for each drug. This feature vector is constructed according to whether there is an association between the drug and the target, and the binary matrix T represents the drug-target association, and T(d(i),t(j)) is set to 1 if the drug d(i) is related to the target t(j), otherwise, it is set to 0. The comprehensive feature vector is a combination of drug substructure feature vectors and drug target feature vectors constructed based on the therapeutic relevance of each drug-target, expressed as:

(1) FV(S(i),T(i))=[S(i)∧T(i)]

**Fig. 2:**
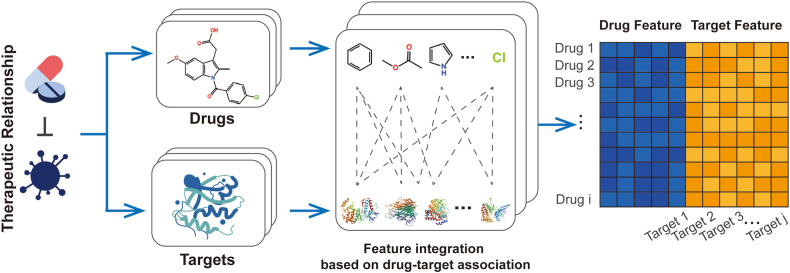
Diagram of feature extraction and integration. Drug substructure features and drug-target association features are integrated into local-global comprehensive features.

S(i) denotes the substructure of the i-th drug that constitutes the substructure feature vector. T(i) is the target feature vector constructed based on the association of the i-th drug with all targets, and ∧ is concatenation. $S\left(i\right)=\left(s_{i1},s_{i2,}s_{i3},\ldots ,s_{ix},\ldots ,s_{iM}\right)$, $T\left(i\right)=\left(t_{i1},t_{i2,}t_{i3},\ldots ,t_{ix},\ldots ,t_{iN}\right)$, $s_{ix}$ is the association between the i-th drug and the substructure at position x, $t_{ix}$ is the association between the i-th drug and the target at position x, and M and N represent the total number of drug substructures and targets in the dataset, respectively. Finally, a comprehensive feature matrix with sample length M+N , and number of samples equal to the number of compounds is obtained. The diagram of feature extraction and integration is shown in Fig. 2.

### 2.3 Deep learning model construction

In order to maximize the usability of our method in the case of an emergency outbreak, we have not only taken into account the reality of limited data, but also tried to use conventional general models in our model construction. This study uses a traditional convolutional neural network model (CNN) to train the data and solve the classification problem.

The convolutional neural network evolved from multi-layer perceptron (MLP) is an artificial neural network mainly developed for image classification [24]. Compared with two-dimensional convolution, one-dimensional convolution is more commonly used in sequence data, signal data, and natural language processing. We proposed a one-dimensional convolutional neural network composed of an input block, two convolution blocks, and one output block.

**Fig. 3:**
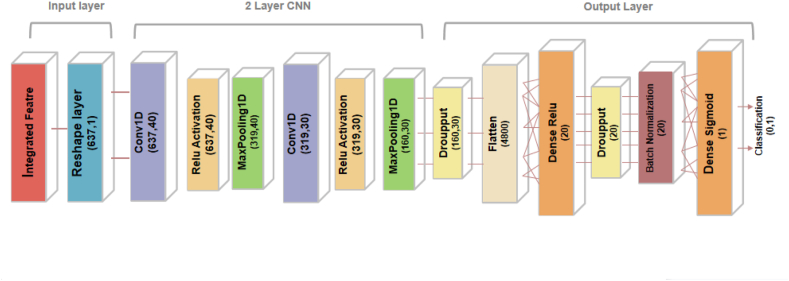
The architecture of the CNN model.

CNN is composed of three basic layers —convolutional layer, subsampling layer, and fully connected layer [25]. In this study, we adopt the traditional CNN architecture. The input block is composed of input data and a reconstruction layer (Reshape). The convolution block consists of a one-dimensional convolution layer, a rectified linear unit (ReLU) [26], and a one-dimensional maximum pooling layer. The output block consists of a Flatten layer, two fully connected layers, and a Softmax layer. After balancing computational efficiency and accuracy, we chose a two-convolution block architecture. The basic architecture of the CNN model is in Fig. 3.

A Reshape layer is placed at the front of the model to convert the input feature vectors into dimensions that the model can identify. Feature extraction and selection are accomplished by cascading convolutional and sampling layers. Forty filters of size 5 are used in the first convolutional layer to perform the convolutional operation, and the number of filters in the second convolutional layer is reduced to 30 to decrease the computational cost and improve the nonlinear capability of the network. ReLU is used as an activation function after the convolutional layer to solve the gradient explosion or vanishing problem and speed up convergence. Feature maps are generated by multiple convolution operations on input samples. The feature mapping $Q_{k}$ of layer k can be described as:

(2) $$Q_{k}=R\left(\;Q_{k-1}⊙W_{k}+b\right)$$

where R denotes the activation function, $W_{k}$ convolution kernel at layer k, b the offset vector, and⊙ the convolution operation. After the convolutional layer, the sampling layer is set to minimize the model parameters, prevent overfitting, and increase the model receptive field. We used maximum pooling in the subsampling layer to preserve the most salient features of each filtered region. The sampling formula $Q_{k}$ can be expressed as:

(3) $$Q_{k}=Maxpooling\left(\;Q_{k-1}\right)$$

The Flatten layer and two fully connected layers using ReLU and Sigmoid as activation functions, respectively, are stacked at the end of the CNN as classifiers to form the output block. Dropout [27] and Batch Normalization (BN) [28] regularization mechanisms are added before the Flatten layer and the last fully connected layer to enhance the stability and robustness of the model. In addition, the L2 regularization penalty is also used in the fully connected layer to keep the weight values small and avoid overfitting. The architecture and detailed parameters of the CNN model proposed in this study are shown in Table 1.

**Table 1:** Detailed parameters of the proposed CNN model.

Type of Layer	Output Shape	Other Parameters
Conv1D	(637,40)	Kernel size = 5, Strides = 1, Filters = 40
Activation	(637; 40)	ReLU activation
Max Pooling 1D	(319; 40)	Pool size = 2
Conv1D	(319,30)	Kernel size = 5, Strides = 1, Filters = 30
Activation	(319; 30)	ReLU activation
Max Pooling 1D	(160; 30)	Pool size = 2
Dropout	(160,30)	Rate 0.5
Flatten	(4800)	–
Dense	(20)	Regularizer L2 (0.01), ReLU activation
Dropout	(20)	Rate 0.8
Batch Normalization	(20)	–
Dense	(1)	Regularizer L2 (0.01), Sigmoid activation

### 2.4 Model training and validation

#### 2.4.1 Training sample division

The feature vector of each drug is composed of the substructure feature vector of the drug and the corresponding target feature vector of the drug. We recorded all the characteristic data of drugs and their targets with potential therapeutic effects from the database as positive samples and assigned the label to 1. The construction of the negative data set is achieved by creating a non-binding drug-target association, with a label of 0. We constructed data sets with the ratio of positive samples to negative samples of 1:1,1:5 and 1:10 respectively and put them into model for learning. We finally select the sample combination with the best training performance for the final prediction model training.

#### 2.4.2 Validation and performance test metrics

To evaluate the performance of LGCNN, we used five-fold cross-validation. One group was selected as the validation set each time, and the remaining groups were used as the training set. The model is fitted on the training set and evaluated on the validation set, repeated five times, with the mean value of the model as the final performance index result. In addition, we also chose to use a separate test set to test the model to obtain more convincing results. Divide the dataset into 90 % for training and 10 % for validation, and make it contain equal numbers of positive and negative samples.

In addition, statistical metrics such as accuracy (ACC), sensitivity (SEN), specificity (SPE), F1 score (F1), positive prediction rate (PPV), negative prediction rate (NPV), Matthew's correlation coefficient (MCC), are employed for further evaluation of the model's performance (See Supplementary method for the formula). The ROC curve was drawn with FPR and TPR as abscissa and ordinate respectively, and the area under the ROC curve (AUC) was calculated. Similarly, the PR curves were plotted with PPV and SPE as horizontal and vertical coordinates, and the area under the PR curve AP was calculated.

Finally, we also compare LGCNN with other classifiers under five-fold cross-validation. The performance of each model was evaluated by the above performance evaluation indexes.

### 2.5 Candidate drug prediction and analysis

#### 2.5.1 Candidate drug prediction

The drug data used for prediction are provided by multiple compound databases, and then the drug-target pairs are further screened with the training sample target range as a constraint. According to the drug-target correspondence, a comprehensive feature vector of candidate drugs was constructed and input into CNN for prediction, and the list of candidate drugs as output. Since the last fully connected layer of CNN uses the Sigmoid activation function, we can obtain the probability that each sample is predicted as a positive and negative sample, respectively. To get more reliable and high-confidence results, we select samples whose probability of being classified as positive is greater than the set threshold for further analysis.

#### *2.5.2* Analysis of prediction results

Since most of the predicted drugs are unlisted small molecule compounds, it is difficult for us to obtain their specific classification information and pharmacological effects. Therefore, the predicted drugs are mainly analyzed from the perspective of bioassay results to study whether they have potential COVID-19 therapeutic effects. Next, the prediction results are analyzed from the perspective of drug substructures, and the presence of each substructure in the superior prediction drug is counted. And search for drugs with a large number of high-frequency substructures among known potential COVID-19 drugs to speculate on the possible functions of predicted repositioned drugs. Finally, the target function of the predicted drug was analyzed to prove the reliability of the prediction results.

### 2.6 Drug-target network and molecular docking simulation

The drug-target interaction network was constructed by integrating the predicted small molecule drugs, targets, and their associated information, and the network was drawn using Cytoscape-3.9.1 [29]. Then the network topology was explored to analyze the role of drugs and targets with high connectivity.

Download the three-dimensional structure of candidate drugs and target proteins. The sdf structure file of the ligand was obtained from PubChem and converted into PDB format using OpenBabel-3.1.1 [30]. The recipient PDB file is obtained from BindingDB and converted to pdbqt format. Then AutoDock-1.5.7 [31] was used to dock the ligand molecules to the active sites of the target protein by a semi-flexible docking method, and the docking binding energy was recorded. PyMOL-2.5.2 [32] was used to visualize the docking results.

## 3 Results

### 3.1 LGCNN demonstrates excellent performance with optimal sample proportion

#### 3.1.1 Sample division and model performance evaluation

**Fig. 4:**
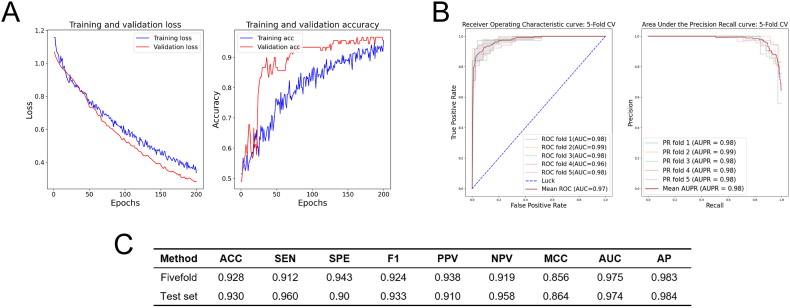
LGCNN performance diagram. (A) Loss values and accuracy change figures of the training set and test set (Positive sample: negative sample = 1:1). (B) ROC and PR curves of the LGCNN. (C) LGCNN cross-validation results based on different datasets.

The LGCNN was trained using datasets with positive and negative sample ratios of 1:1, 1:5, and 1:10, and the classification results were verified by the verification set. The hyperparameters of the model are set as follows: epoch is set to 200, Adam optimizer is used and the learning rate is set to 0.0001, and the batch size is set to 30. It was observed that LGCNN exhibited the best convergence when the ratio of positive and negative samples was 1:1. Therefore, this dataset was utilized to train the model. The distribution of the loss function and accuracy during model training, with positive and negative sample ratios of 1:1, 1:5, and 1:10, is shown in Fig. 4A, Fig. S1, and Fig. S2 respectively.

The average area under the ROC curve (AUC) was 0.975, and the average area under the PR curve (AP) was 0.983. The model evaluation indicators under five-fold cross-validation and an independent test set are depicted in Fig. 4C. Additionally, the area under the precision-recall curve was calculated as an additional performance measure for the model. The ROC curve and PR curve of the five-fold cross-validation are shown in Fig. 4B.

#### 3.1.2 Comparison with different classifiers

To further assess the efficacy of LGCNN, we compared it with three state-of-the-art classifiers: random forest (RF) [33], support vector machine (SVM) [34], and Light Gradient Boosting Machine (LightGBM) [35], and two deep learning models: Long Short-Term Memory(10.13039/100014976LSTM) [36] and Deep Residual Shrinkage Networks(DRSN) [37] under five-fold CV. The comparison models were adjusted based on parameters and trained and tested on the dataset used in the study. The experimental results are shown in Table 2, and the bold value indicates the best performance.

**Table 2:** Comparison of LGCNN with the competing classifiers under the fivefold CV experiment.

Method	ACC	SEN	SPE	F1	PPV	NPV	MCC	AUC	AP
SVM	0.870	0.854	0.880	0.867	0.883	0.860	0.738	0.922	0.943
LightGBM	0.850	0.867	0.826	0.851	0.840	0.868	0.700	0.917	0.908
RF	0.830	0.718	0.942	0.807	0.925	0.768	0.676	0.924	0.938
LSTM	0.845	0.749	**0.949**	0.833	**0.946**	0.792	0.717	0.869	0.913
DRSN	0.854	0.883	0.821	0.858	0.839	0.878	0.711	0.933	0.934
LGCNN	**0.928**	**0.912**	0.943	**0.924**	0.938	**0.919**	**0.856**	**0.975**	**0.983**

**Fig. 5:**
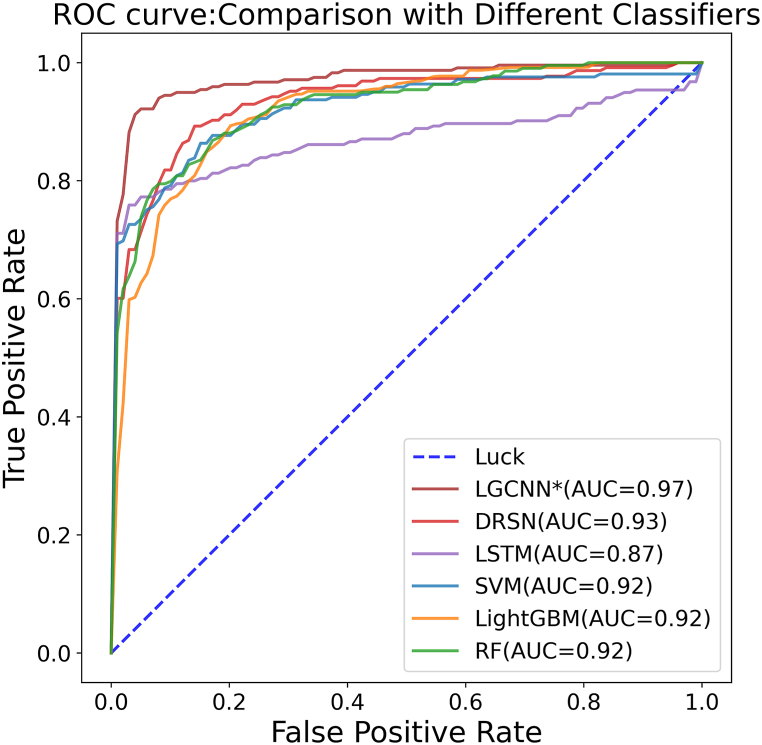
ROC curves obtained by LGCNN and other competing methods on the limited datasets based on 5-fold CV.

The experimental results demonstrate that LGCNN outperforms the other models in most of the evaluation index. Specifically, the AUC and AP of LGCNN are 4.50 % and 4.24 % higher than the corresponding second-best results, respectively. The ROC curves of different classification methods are shown in Fig. 5. The AUC value of the LGCNN model is 0.975, while the AUC values of SVM, LightGBM, random forest, LSTM, and DRSN are 0.922, 0.917, 0.924, 0.869, and 0.933 respectively.

Notably, consistent with the expected results, the two neural network models used for comparison, LSTM, and DRSN, outperformed traditional machine learning models on most performance metrics. However, some indicators of the LSTM model do not show better performance, and even the AUC is lower by comparison. This may be because LSTM, as a variant of recurrent neural network (RNN) [38], requires more parameters to participate in training, has higher computational complexity, and may have insufficient generalization ability when faced with limited data. As an improved convolutional neural network model, DRSN has not shown higher performance, which may be due to the introduction of residual units increasing the complexity of the model, and instead making its performance on datasets with simple features lower than that of simple CNN.

In conclusion, although LGCNN is an improvement based on traditional CNN, its advantage is that it maintains low complexity through local connections and weight sharing. By accurately capturing the features of drug chemical structures and drug-target connections, it shows excellent performance on limited datasets.

### 3.2 Prediction and analysis of predicted potential therapeutic drugs for COVID-19

#### 3.2.1 Bioassay results confirm the potential of the high-scoring predicted drug as a treatment for COVID-19

After training, evaluation and validation, LGCNN were applied to screen for therapeutic potential from 210,841 compounds from drug and small molecule databases. The samples were assigned a probability score indicating possible therapeutic effects, which served as a scoring index in this study to identify potential COVID-19 therapeutic drugs. Based on these scores, 208 drug-target combinations were identified as excellent combinations. The top 25 % of drugs were selected from 208 drugs for further analysis. As for the structural classification of known compounds, 31 out of the 52 drugs belonged to the class of fluorinated aromatic substances, with 2 being piper-azinyl norbenzomorphones, 2 being dibenzocycloheptanes, 1 being a cysteine-type sesquiterpene lactone, 1 being a tricyclic heteroaryl compound, 2 being oxydiazepine compounds, 1 being a pyrazole derivative, 1 being a carboxylic acid, and 11 being other types of small molecule compounds. From a functional perspective, the analysis of compound functions revealed that most of the 52 high-scoring drugs possessed noteworthy functions, including mitogen-activated protein kinase (MAPK) antagonists, tumor necrosis factor-α (TNF-α) antagonists, cathepsin K (CTSK) antagonists, dihydroorotate acid dehydrogenase (DHODH) antagonists, histone deacetylase (HDAC) antagonists, and tyrosine-protein kinase JAK2 (JAK2) antagonists. In PubChem assay presentation, compounds with any of Ki, IC50, Kd, or EC50 activity value ≤ 10uM are labeled as “Active". Table 3 lists some of the notable inhibitors among the predicted compounds and their relevant information.

**Table 3:** Bioassay result of top 25 % compounds (portion).

Compound	Structure	Target Name	BioAssay Name	Activity Type	Activity Value (μM)
CHEMBL549630		MAPK14	Inhibition of human p38alpha by FRET assay	IC50	0.038
		TNF-α	Inhibition of LPS-induced TNFalpha release in human THP1 cells after 5 h by ELISA	IC50	0.211
		TNF-α	Inhibition of LPS-induced TNFalpha production in human whole blood after 24 h by ELISA	IC50	0.47
CHEMBL358548		MAPK14	In vitro inhibitory concentration against Mitogen-activated protein kinase p38 alpha	IC50	0.086
CHEMBL473984		MAPK14	Inhibition of p38alpha MAPK by cell-free enzyme immunosorbent assay	IC50	1.08
CHEMBL473598		MAPK14	Inhibition of p38alpha MAPK by cell-free enzyme immunosorbent assay	IC50	0.44
CHEMBL475361		MAPK14	Inhibition of p38alpha MAPK by cell-free enzyme immunosorbent assay	IC50	2.73
CHEMBL200004		CTSK	Inhibitory activity against human cathepsin K using Z-Leu-Arg-NHMec substrate	Ki	6.5
CHEMBL2263447		DHODH	Inhibition of Homo sapiens (human) dehydroorotate dihydrogenase using 2,6-dichlorophenol-indophenol as substrate after 10 min by spectrophotometric analysis	IC50	0.005
		DHODH	Inhibition Assay from US Patent US8691852: “Amino nicotinic and isonicotinic acid derivatives as DHODH inhibitors"	IC50	0.005
CHEMBL3617543		HDAC6	Inhibition of HDAC6 (unknown origin) using RHKK(Ac) fluorogenic acetylated peptide substrate by fluorometric assay	IC50	0.15
		HDAC8	Inhibition of HDAC8 (unknown origin) using RHK(Ac)K(Ac) fluorogenic acetylated peptide substrate by fluorometric assay	IC50	3.3
CHEMBL603432		JAK2	Inhibition of JAK2 by radiometric assay	Ki	2
CHEMBL477190		MAPK14	[4-(4-Cl-3-hydroxyphenyl)-5-(4-pyridinyl)-1H-imidazole-2yl]phenyl}methyl)amino]carbonyl}benzoic acid from human p38alpha by fluorescence polarization	Ki	0.0006

Note: From the first column to the sixth column, compound name, compound 2D structure, biological detection target name, bioassay name, activity type, and activity value are listed respectively.

Notably, out of the top 25 % of drugs, there were 24 members of the fluorinated aromatic aminopyridine N-oxide family, which possessed non-fluorinated aliphatic side chains. These drugs demonstrated efficient and selective inhibition of p38α MAPK and had a balanced efficacy and pharmacokinetics. Most of the aminopyridine N-oxide family members exhibited inhibition of both MAPK and TNF-α, indicating their potential therapeutic relevance for COVID-19. For instance, CHEMBL549630, the highest-scoring compound, showed excellent inhibitory ability against MAPK14 and TNF-α. Literature has suggested that MAPK and TNF-α are potential therapeutic targets for COVID-19 [[39], [40], [41], [42]] Another high-scoring compound CHEMBL200004 displayed an inhibitory activity against CTSK in biological detection. CTSK has been proposed as a potential target for the treatment of COVID-19 [43]. Similarly, the predicted high-scoring compounds CHEMBL2263447, CHEMBL3617543, and CHEMBL2600919 exhibited inhibition of DHODH, HDAC, and JAK2 respectively in biological detection experiments. Moreover, DHODH, HDAC, and JAK2 are considered noteworthy targets in COVID-19-related research [[44], [45], [46]]. Particularly, the predicted drug Parthenolide, a sesquiterpene lactone found in the herb Piju, belongs to a non-steroidal anti-inflammatory agent in pharmacological classification. Studies have indicated that Parthenolide's anti-hepatitis C virus (HCV) activity [47], also has a positive effect on the treatment of COVID-19 [48,49]. Based on the analysis of high-scoring predicted compounds' biological activity, it can be concluded that these compounds have excellent therapeutic potential for COVID-19.

#### 3.2.2 Substructural analysis reveals the therapeutic potential of the predicted drugs

A high-frequency substructure set was derived from the substructures of the 208 drugs (see the 'Analysis of prediction results' in the method section). Many drugs with these high-frequency substructures were found among the known potential COVID-19 drugs (training data), indicating their relevance. Sorting the drugs based on the number of high-frequency substructures, we analyzed the top 10 drugs (Table 4), which included 4 anti-infective agents, 2 anti-tumor agents, 2 enzyme inhibitors, 1 immunotherapy agent, and 1 antiplatelet agent. Notably, posaconazole and itraconazole, both effective triazole antifungal agents used in the prevention of invasive fungal infections caused by aspergillosis and Candida in high-risk patients, were mentioned in many studies for the prevention and treatment of COVID-19-related complication mucormycosis [50,51]. Moxifloxacin, an FDA-approved antibacterial prescription drug primarily used to treat certain bacterial infections, has demonstrated potent antiviral activity and immunomodulatory properties in studies, making it a potential drug for adjuvant treatment of COVID-19. Both papilmod and dasatinib are protein kinase inhibitors commonly employed in the treatment of rheumatoid arthritis and chronic myelogenous leukemia, respectively. Some studies have proposed their repositioning for the treatment of COVID-19 [52,53]. Ticagrelor has shown the potential therapeutic effect on coagulation dysfunction caused by COVID-19-related sepsis [54] and a strong binding ability with the spike protein and membrane protein of SARS-CoV-2, which indicates its promise as a COVID-19 treatment drug [55,56].

**Table 4:** Information on known potential COVID-19 treatments rich in high-frequency substructures.

Rank	Drug name	Classification	PMID
1	Telacebec	antibacterial agent	–
2	Posaconazole	Antifungal agent	35390293,5220559
3	Abivertinib	antineoplastic agent	35136710,823185
4	Danicopan	Immunotherapeutic agent	–
5	Sporanox	antibacterial agent	35064041,984948
6	Ticagrelor	anti-platelet agents	32445671,510337
7	Subasumstat	antineoplastic agent	–
8	Moxifloxacin	antibacterial agent	32546446 33063271
9	Pamapimod	enzyme inhibitor	34537409
10	Dasatinib	enzyme inhibitor	34009288,517409

Note: The first to fourth columns are drug ranking, drug name, drug type, and related literature Pubmed ID (' - ' indicates that there is no supportive study).

Assessing the function of these high-frequency substructure-rich drugs, it can be inferred that drugs containing these substructures have the potential to treat or assist in the treatment of COVID-19 and its complications. Therefore, drugs predicted by extracting these substructures may also possess similar functions. In summary, substructure analysis reveals the excellent disease treatment performance of the predicted drugs, and this substructure-based drug recognition method provides a potential avenue for rapid identification of therapeutic drugs for unknown pathogens.

### 3.3 Analysis of target functions explaining the potential therapeutic mechanism of the predicted drug

The integration of targets for the predicted compounds resulted in a set of 23 targets, which were analyzed to determine their functions. This analysis provided evidence that some of these targets are associated with COVID-19. The information related to these targets, along with the supporting literature, is organized in Table S3, and it can be observed that most of the predicted compounds are associated with COVID-19.

#### 3.3.1 Potential targets for blocking SARS-CoV-2 infection

Through a literature search, it was discovered that certain targets are involved in the process of viral infection, presenting potential opportunities for drug intervention. For instance, cathepsin B (CTSB) has been shown to participate in the activation and cleavage of spike proteins during the entry of SARS-CoV-2 into human hosts [57,58]. Additionally, sigma non-opioid intracellular receptor 1 (SIG-1R) has been found to regulate the budding or release of the coronavirus [59]. Tyrosine kinase Abl1 (ABL1) can also regulate the fusion and release of coronavirus spike proteins [60,61], with several COVID-19 studies mentioning its potential therapeutic effects [62,63]. Blocking these targets with antagonists may achieve targeted inhibition of SARS-CoV-2 by impeding the viral infection process, thereby reducing the damage caused by the virus to the host.

#### 3.3.2 Potential targets for alleviating inflammatory response

Certain targets have been identified to be associated with the inflammatory response triggered by SARS-CoV-2 invasion. For instance, MAPK14 and MAPK8 are members of the MAPK family, and the upregulation of the p38 MAPK pathway activates pro-inflammatory cytokines [64] playing a role in the development of inflammatory damage caused by SARS-CoV-2 [39]. Investigations have suggested that MAPK14 and MAPK8 should be considered as potential new targets for COVID-19 treatment [[65], [66], [67], [68]]. Clinical data has demonstrated that TNF is commonly present in the blood and diseased tissues of COVID-19 patients, with its levels increasing as the disease progresses [69,70]. TNF-α, being a pro-inflammatory cytokine, selectively targets lung tissue and its overproduction can significantly worsen prognosis [71]. Some studies have proposed to consider the potential therapeutic role of TNF inhibitors for COVID-19 [41,42].

Since SARS-CoV-2 infection triggers a cytokine storm, leading to severe organ damage, known as acute respiratory distress syndrome (ARDS) [72], it is crucial to address the inflammation-induced damage. Elevated pro-inflammatory cytokines leading to chronic inflammation are also observed as a symptom of long-COVID [73]. The use of anti-inflammatory drugs, particularly for neuroinflammatory-induced cognitive impairment syndrome, is beneficial for long COVID treatment [74].

#### 3.3.3 Potential targets for multi-pathway COVID-19 treatment

Certain targets are associated with both the processes mentioned above and are considered to have a more significant role in the treatment of COVID-19. JAK1 and JAK2, non-receptor tyrosine kinases involved in multiple stages of cell development and epigenetic modification processes, impact the inflammatory process and virus entry events in COVID-19 [75]. JAK inhibitors such as Fedratinib, Baricitinib, Ruxolitinib, and Tofacitinib have been clinically used to treat cytokine storms caused by SARS-CoV-2 [46,[76], [77], [78]]. DHODH, an important flavin-dependent mitochondrial enzyme in pyrimidine de novo synthesis, has been proposed as a target for anti-SARS-CoV-2 therapy due to its dual role in inhibiting viral genome replication and regulating the immune system [44,79]. Targets associated with COVID-19 through multiple pathways are more likely to play a key role in clinical treatment, and drugs designed based on these targets are more likely to have higher efficacy.

Based on the analysis above, the predicted candidate small molecule targets have potential in the treatment of COVID-19. These targets are located in host cells and are involved in the SARS-CoV-2 infection process or (and) inflammatory damage pathways. Therefore, it can be inferred that these potential drugs may alleviate symptoms by reducing the inflammatory response and addressing the root cause by inhibiting virally infected cells. Targets involved in multiple processes are believed to play a more significant role in the disease's development, and the corresponding drugs are expected to have greater therapeutic potential.

### 3.4 Roles of highly connected nodes in the drug-target network for COVID-19 treatment

**Fig. 6:**
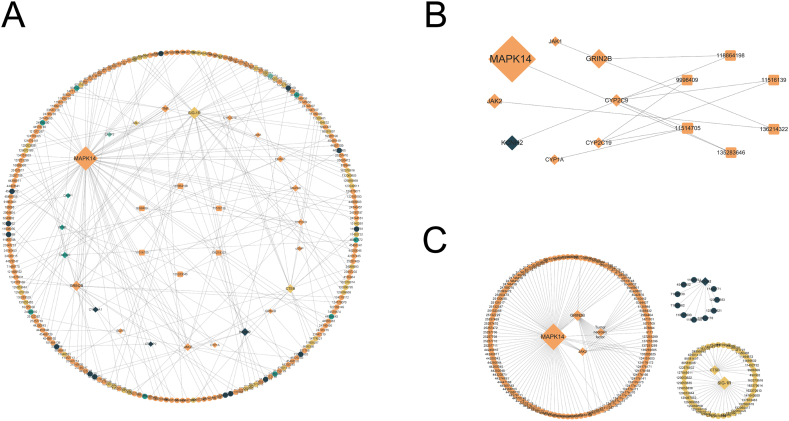
Drug-target interaction network. (A) Complete drug-target interaction network predicted by LGCNN. (B) Drug-target interaction subnetwork for multi-target drugs (cid). (C) Drug-target interaction subnetworks for targets modulated by multiple drugs (cid).

The associations between 208 small molecule drugs and their targets were compiled to construct a drug-target network, which was visualized using Cytoscape. The network was then analyzed to calculate the connectivity of nodes, emphasizing drugs and targets with high connectivity (Fig. 6A). In this network, targets, single-target drugs, and multi-target drugs are denoted by diamond, circle, and square dots, respectively. The colors assigned to the targets and drugs represent their potential associations with COVID-19: orange indicates an association with the COVID-19 inflammatory process, yellow indicates an association with the SARS-CoV-2 infection process, green indicates an association with both, and blue indicates no association found.

Through the calculation of node connectivity in the network, five multi-target drugs were identified: CHF5074, SCHEMBL17493825, CHEMBL372751, CHEMBL398126, and SCHEMBL14708271 (Fig. 6B). The drug CHF5074, a novel γ-secretase regulator, is commonly used to treat Alzheimer's disease [80], transmissible spongiform encephalopathy [81], and hereditary amyloid polyneuropathy [82]. Patent information from the World Intellectual Property Organization (WIPO) indicates that SCHEMBL17493825 belongs to pyrazole derivatives, which can be used as an NMDA NR2B receptor inhibitor to treat central nervous system diseases; Similarly, CHEMBL372751, a 1-phenyl alkanoic acid derivative, is involved in the treatment of Alzheimer's disease and multiple sclerosis. CHEMBL398126, a phenoxy acid is linked to the treatment of neuromuscular diseases. SCHEMBL20448745, a benzamide derivative, acts as a p38 MAPK inhibitor in preventing and treating diseases mediated by p38 kinase activity. Furthermore, SCHEMBL14708271, a tricyclic heterocyclic compound, functions as a JAK inhibitor to prevent and treat autoimmune, inflammatory, and allergic diseases. These drugs have multiple targets in the network. LGCNN enables the prediction of multi-target drugs because the model transfers the mapping of global features based on therapeutic mapping between drugs and multi-targets into local maps of substructural features and target features. Generally, multi-target drugs exhibit better efficacy and safety compared to single-target drugs while also presenting a lower risk of drug-drug interaction.

In terms of connectivity ranking, the top 5 targets in the network are MAPK14, SIG-1R, GluN2B, CTSB, JAK2, TNF-α, and KCNH2 (JAK2, TNF-α, KCNH2 have the same connectivity) (Fig. 6C). Many of these targets have been previously identified as potential therapeutic targets for COVID-19 in other studies. It's noteworthy that targets with high connectivity have a similar role in participating in the treatment of COVID-19. The activation of MAPK14, JAK2, and TNF-α is related to the inflammatory response. CTSB and SIG-1R are involved in viral entry into host cells and replication [57,83]. Most of the predicted candidate drugs target these various pathways. Therefore, the commonness and characteristics of highly connected targets mirror the characteristics of drugs used in the treatment of COVID-19. This suggests that the drugs predicted by LGCNN may possess host-oriented characteristics, interfering with the entry of SARS-CoV-2 into host cells or impacting its RNA replication process. Moreover, these drugs may alleviate damage to other organs caused by the cytokine storm resulting from SARS-CoV-2 invasion by antagonizing proinflammatory cytokines.

### 3.5 Molecular docking simulation identified drug-target interactions with high affinity

To confirm the interaction between the top 25 % of small molecule compounds and the target protein, we employed AutoDock for molecular docking simulation. AutoDock conducted ten docking attempts per ligand-receptor pair, and the best results were selected for final analysis. Table 5 presents the docking scores for the top 10 small molecule drugs and targets.

**Table 5:** Predicted molecular docking results of small molecule drugs (top 10).

Rank	Ligand	Structure	Receptor	Binding energy (kcal/mol)
1	CHEMBL2263447		DHODH	−10.15
2	SCHEMBL18785395		SIG-1R	−10.09
3	CHEMBL473984		MAPK14	−9.83
4	CHEMBL474178		MAPK14	−9.67
5	CHEMBL218961		MAPK14	−9.6
6	CHEMBL375978		MAPK14	−9.57
7	SCHEMBL18785392		SIG-1R	−9.53
8	CHEMBL447190		MAPK14	−9.24
9	CHEMBL475361		MAPK14	−8.98
10	CHEMBL514321		MAPK14	−8.74

Note: The first to fifth columns are the affinity ranking, ligand name and 2D Structure, ligand structure, receptor name, and docking binding energy.

The binding energy, which represents the energy released during receptor-ligand binding, serves as a general indicator of affinity. Typically, binding energies less than −1.2 kcal/mol or −5 kJ/mol are considered feasible for docking results. The results of candidate drug-target molecular docking showed that the binding energies of the high-score small molecule compounds and their targets showed excellent affinity. It is worth noting that the top 10 receptors in the molecular docking results are mitogen-activated protein kinase 14 (MAPK14). Previous studies have identified MAPK14 as a potential target for COVID-19 treatment [66,84], as MAPK inhibitors have demonstrated promise in mitigating host damage caused by virus-induced inflammatory responses [39].

**Fig. 7:**
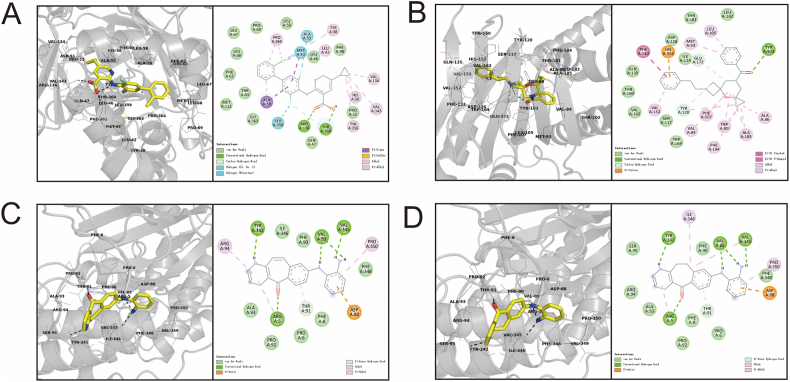
Virtual docking diagrams of ligands and receptors. 3D interactions of ligand-receptor complex (left) and 2D interactions of ligand-receptor complex (right) in each subdiagram. (A) CHEMBL2263447-DHODH docking complex. (B) SCHEMBL18785395-SIG1R docking complex. (C) CHEMBL473984-MAPK14 docking complex. (D) CHEMBL474178-MAPK14 docking complex.

To gain insight into the binding mechanism of the screened compounds with their targets, we conducted the three-dimensional visualization of the ligand-receptor complexes using PyMOL and the two-dimensional visualization using Discovery Studio (DS). The binding diagram of the four example compounds is shown in Fig. 7, and the interactions between receptors and ligands are summarized in Table S4.

The molecular docking results validate the reliability of our predicted high-scoring drugs from an energetic perspective, exhibiting a strong affinity towards the potential therapeutic target of COVID-19.

### 3.6 Drug-mechanism-target analysis revealed the possible universal mechanism of anti-coronavirus drugs

**Fig. 8:**
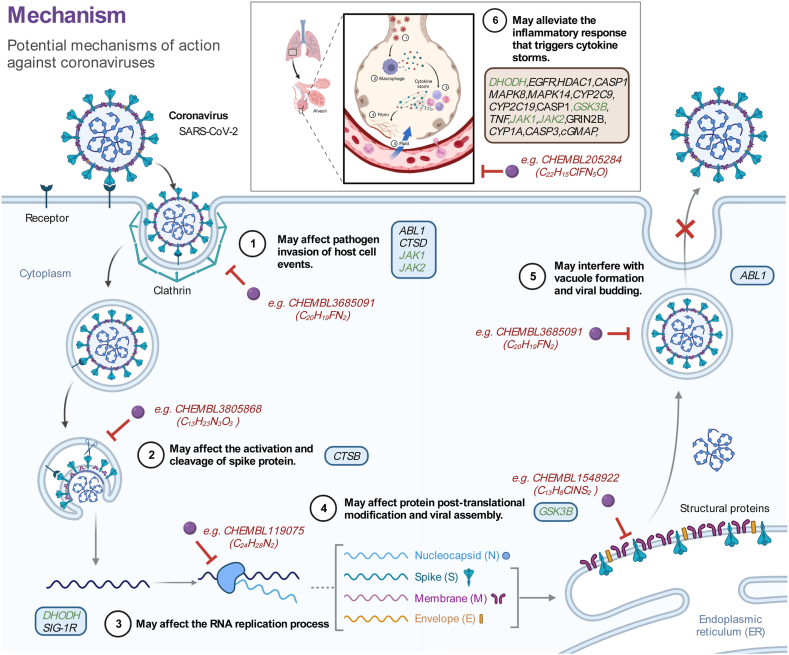
Compound-mechanism-target diagram. This diagram illustrates the possible pathways through which high-scoring predictive drugs may treat COVID-19 via target engagement. Each target is assigned a serial number. Targets involved in viral infection replication processes are indicated by blue boxes, while targets implicated in the inflammatory response triggered by viral invasion are marked with brown boxes. Targets involved in both processes are labeled green. The example compound CHEMBL ID and the chemical formula corresponding to each pathway are highlighted in red. (For interpretation of the references to color in this figure legend, the reader is referred to the Web version of this article.)

Finally, we constructed a compound-mechanism-target diagram based on the pathways in which these targets are implicated in COVID-19, elucidating the potential mechanisms of action of predicted drugs (Fig. 8). With consideration of the coronavirus life cycle and the inflammatory response triggered by viral invasion, we propose six potential pathways through which these compounds may exert their effects. Including affecting pathogen invasion of host cell events, affecting the activation and cleavage of spike protein, affecting the RNA replication, affecting protein post-translational modification and viral assembly, interfering with vacuole formation and viral budding, and alleviating the inflammatory response that triggers cytokine storms. Existing studies have provided supportive evidence on the feasibility of these mechanisms [85,86]. These potential mechanisms provide a comprehensive treatment strategy for COVID-19, targeting both its symptoms and underlying causes. Importantly, these mechanisms are not specific to COVID-19 and may offer a universal regimen against other HCoVs (SARS-CoV, MERS-CoV, SARS-CoV-2).

## 4 Discussion

In view of the experience and lessons learned from the COVID-19 pandemic, there is an urgent need for an effective method to screen antiviral drugs and address the potential recurrence of infectious disease pandemics in the future. Our study presents an integrated deep learning architecture that combines drug substructure characterization and drug-target interaction features at the local and global levels based on limited coronavirus therapy-related data to infer promising preclinical drug candidates for the treatment of SARS-CoV-2 infection.

One of the reasons for the decent results is that LGCNN is able to maximize the information obtained from both the local and global levels, respectively. In LGCNN, the local features are represented by the mapping associations between the MACCs substructural features of the drug and the target. Global features are reflected in three different aspects: First, beyond the limitation of focusing only on drug structure, drug-target interaction information is incorporated to construct drug-disease-target associations at a holistic level; Second, different from the traditional model design based on the 3D structure and binding sites of individual therapeutic targets, LGCNN constructs the global mapping associations between drugs and multiple targets based on therapeutic effects, which is the key to construct the local mapping between substructure features and target features. Multi-target compounds show superior efficacy and safety in the clinic with a lower risk of drug-drug interactions; Third, drug-target features are integrated and embedded into a convolutional neural network, which is mapped after a multilayer convolutional cascade to the overall features of potential COVID-19 therapeutic drugs. Although not in the same field, Wang et al. also confirmed that the whole-local combination is one of the most optimal feature representations in the case of limited data [87].

In this study, we used CNN to achieve feature extraction and classification rather than cumbersome feature representations and complexly structured models. This is because researchers are unlikely to collect a large variety of features in the early stages of an emerging epidemic, and a simple and lightweight model structure is also more suitable for the re-emergence of Disease X. On the whole, the performance of our model is superior to traditional machine learning methods and general deep learning models. With the further research, many high-precision, feature-rich, and structurally complex drug prediction screening models have emerged [88,89]. For example, Su et al. [90] proposed a deep learning method, VDA-DLCMNMF, based on the constrained multi-view nonnegative matrix factorization (CMNMF) model for learning association matrices based on drug-virus relationships between drug chemical structures and viral genome sequences to achieve drug repositioning. Zhao et al. [91] developed an improved graph representation learning method, iGRLDTI, to discover novel drug-target interactions based on heterogeneous biological information network. In comparison, LGCNN may be more applicable in the early stages of a disease outbreak with limited data and ambiguous features.

Our findings revealed that the predicted highly scored compounds may achieve disease remission by acting on specific targets to effect either a blockage of SARS-CoV-2 proliferation to reduce viral infectivity or (and) a reduction in SARS-CoV-2-induced inflammatory damage. These agents may be used alone or in combination to combat the etiology and symptoms of COVID-19. However, it is worth noting that these mechanisms are not unique to COVID-19. Comparison with previous treatments of SARS and MERS reveals that a significant portion of these mechanisms is universal. For example, cathepsin inhibitors are used to inhibit the cleavage process of S protein after viral endocytosis [92,93], and Abelson kinase inhibitors are used to block coronavirus virion fusion with the endosomal membrane, thereby inhibiting viral replication [62,94]. In addition, viral attacks can cause a systemic inflammatory response in the host, which can cause damage to various tissues and organs, leading to multiple organ failure and even death. Anti-inflammatory drugs or immunosuppressive agents are commonly used to alleviate the damage caused by cytokine storms [95]. A comprehensive study integrating and comparing transcriptome data from three pathogenic HCoVs (SARS-CoV, MERS-CoV, SARS-CoV-2) identified the activation of common pathways related to coronavirus infection, including the JAK-STAT signaling pathway, TNF signaling pathway, and MAPK signaling pathway [96], which were also identified in our study. In general, drugs that counteract the pathogenic characteristics of HCoV will be more effective in treating coronavirus-infected diseases.

Design strategies for broad-spectrum antiviral drugs (BSA) typically fall into two categories: host-targeted antiviral drugs (HTA) and direct-acting antiviral drugs (DAA) [97]. However, when a new virus emerges, its specificity often results in a longer development timeline for specific DAA. In contrast, HTA has better advantages in drug resistance and universality by blocking viral replication to overcome potential viral mutations [98]. Targeting the targets involved in the host infection process rather than the traditional single inhibition based on viral proteins led to the improved generalization performance of the drug screening method proposed in this study against coronaviruses.

At the same time, there are some limitations in the current study. The negative samples used in this study were created by randomly selecting samples from unproven drug-target relationships. Even though the probability is low, there may be errors caused by false negatives due to unrecognized positive interactions. In addition, the number of known potential drug-target associations is limited, resulting in a relatively weak number of training samples. In summary, LGCNN may achieve rapid screening of excellent anticoronaviral drugs, but there is still room for further improvement. First, a reasonable solution should be set up to address the above limitations. Second, integrate more features to extend the precision, such as topological information and physicochemical properties of ligands. Finally, we would like to further evaluate the generalization ability of the model by applying it to other diseases or other prediction problems.

## 5 Conclusions

This study presents a novel deep learning framework for drug screening, LGCNN, which is capable of rapidly identifying potential therapeutic approaches against pandemic viral infections by utilizing local-global features of drugs, targets, and their associations Applied to COVID-19, 208 potential therapeutic compounds were screened from 210,696 compounds based on features integrated from limited clinical trial data. LGCNN enables the discovery of multi-target therapeutics as well as therapeutics with novel holistic structures by identifying mapping relationships between local drug substructures and targets under conditions of global relevance of therapeutic effects. Substructure analysis revealed the model's ability to identify structurally diverse compounds, while target function analysis confirmed their potential to inhibit viral infection and attenuate inflammatory responses. In addition, the simple structure with underlying feature selection improves the generalizability of LGCNN for rapid drug screening. In conclusion, this framework offers a valuable tool for data-driven drug discovery, particularly in the context of emerging diseases where information is limited.

## Funding

This study was supported by the 10.13039/501100001809National Natural Science Foundation of China (62072144, 61671191) and the Heilongjiang Postdoctoral Initiation Grant (LBH-Q20159).

## Data availability statement

All data in this study are publicly available data. This paper does not report original code. Data related to the main results of the article can be found in the supplementary materials. Further information and requests for resources should be directed to and will be fulfilled by the lead contact.

## CRediT authorship contribution statement

**Ying Wang:** Writing – original draft, Validation, Software, Data curation, Conceptualization. **Yangguang Su:** Writing – review & editing, Validation, Software, Methodology, Data curation. **Kairui Zhao:** Writing – review & editing, Visualization, Validation, Formal analysis, Data curation. **Diwei Huo:** Writing – review & editing, Software, Data curation, Conceptualization. **Zhenshun Du:** Writing – review & editing, Visualization, Validation. **Zhiju Wang:** Writing – review & editing, Visualization, Validation. **Hongbo Xie:** Writing – review & editing, Visualization, Validation. **Lei Liu:** Writing – review & editing, Visualization, Validation. **Qing Jin:** Writing – review & editing, Visualization, Validation. **Xuekun Ren:** Writing – review & editing, Supervision, Methodology, Formal analysis, Conceptualization. **Xiujie Chen:** Writing – review & editing, Supervision, Project administration, Methodology, Funding acquisition. **Denan Zhang:** Writing – review & editing, Project administration, Methodology, Funding acquisition, Conceptualization.

## Declaration of competing interest

The authors declare that they have no known competing financial interests or personal relationships that could have appeared to influence the work reported in this paper.
