# Supplementary material for: A deep learning drug screening framework for integrating local-global characteristics: A novel attempt for limited data
Source: Heliyon. 2024 Jul 14;10(14):e34244. doi: 10.1016/j.heliyon.2024.e34244 (PMC11315141; doi:10.1016/j.heliyon.2024.e34244)
Supplement: Multimedia component 1 [file mmc1.docx]

Supplementary Methods

**Data screening**

The potential COVID-19 therapeutic drugs and target data used in this study were from PubChem ([Kim et al., 2021](#_ENREF_5)), Drugbank ([Wishart et al., 2018](#_ENREF_11)), BindingDB ([Gilson et al., 2016](#_ENREF_3)), and TTD ([Zhou et al., 2022](#_ENREF_12)) databases. Among them, 548 compounds were obtained from PubChem database, 68 from Drugbank database and 138 from TTD database. The experimental stage and target information of SARS-COV-2 treatment drugs were also obtained from the BindingDB protein-small molecule interaction database. In addition, since some of the potential therapeutic drugs recorded in the PubChem and Drugbank databases have no clear targets, all small molecules and drug targets are obtained from the BindingDB database for drug target correspondence. At the same time, 338 drugs with SMILES ([Öztürk et al., 2016](#_ENREF_9)) structure and target information were extracted, and 5211 drug-target interactions. After preliminary screening, we re-screened their target types for the drug-target correspondence, considering that these targets have some effects on other organisms. Based on the process of neo-coronavirus infection of human cells, we can infer that the targets of potential anti-neo-coronavirus drugs are located in the virus or the human body. Therefore, we screened the drug targets and ended up with 223 drugs, 470 targets, and 1036 drug-target interactions.

The candidate drug structure and target data for prediction were obtained from Drugbank ([Wishart et al., 2018](#_ENREF_11)), TTD ([Zhou et al., 2022](#_ENREF_12)), pharmGKB ([Whirl-Carrillo and Huddart, 2021](#_ENREF_10)), PubChem ([Kim et al., 2021](#_ENREF_5)), DGIdb ([Cotto et al., 2018](#_ENREF_1)), BioGRID ([Oughtred et al., 2019](#_ENREF_8)), and BindingDB ([Gilson et al., 2016](#_ENREF_3)) databases. Drugs and small molecule compounds with SMILES structure and target information were extracted, of which 1253 were from the Drugbank database, 22,915 drugs from the TTD database, 794 drugs from the pharmGKB database, 9375 drugs from the DGIdb database, 3829 species from the BioGRID database, and 463837 drugs from the BindingDB database. Based on the above databases, we obtained a total of 495295 drugs and small molecules. Similarly, virus and human sources were screened for the targets of these compounds. In particular, the targets of these compounds must be 470 targets of potential COVID-19 therapeutic drugs, so that their target sources are consistent with the sources of potential anti-COVID-19 therapeutic drug targets. A total of 210696 drugs and small molecules were obtained, of which 301785 drug-target interactions were obtained.

**Drug and target representation**

The molecular access system fingerprints (MaccsKeys) ([Durant et al., 2002](#_ENREF_2)) (<https://github.com/rdkit>)in the Python programming package RDKit were used to extract drug substructure feature vectors from the SMILES sequence.MaccsKeys are generally utilized in drug similarity calculation, drug structure-activity relationship prediction, and protein binding pocket coding, which contains 166 substructural features, each position encoding a substructure. The substructure feature vectors of all compounds form the drug substructure matrix $S$. If the drug $d\left( i \right)$ has a substructure $s\left( j \right)$, then $S\left( d\left( i \right),s\left( j \right) \right)$ is set to 1, otherwise 0. We constructed a target feature vector based on the therapeutic association for each drug. This feature vector is constructed according to whether there is an association between the drug and the target, and the binary matrix $T$ represents the drug-target association and $T\left( d\left( i \right),t\left( j \right) \right)$ is set to 1 if the drug $d\left( i \right)$ is related to the target $t\left( j \right)$,otherwise, it is set to 0.

**Architecture of the CNN model**

To change the dimension of the multidimensional matrix while keeping the number of elements unchanged, the model first uses a Reshape layer to convert the input feature vector into a dimension that the model can identify. Then, the cascade convolution layer and sampling layer are used to set up to extract and select features to achieve better results. The classification effect of the CNN model is very dependent on the number and size of the filter. As the size of the filter and the number of filters change and the performance of the CNN will also change. At the first convolutional layer, we use 40 filters of size 5 to perform convolution operations. The number of filters at the second convolution layer is reduced to 30 to reduce the computational cost and improve the nonlinear ability of the network. We use ReLU ([Nair and Hinton, 2010](#_ENREF_7)) as the activation function after the convolution layer to solve the gradient explosion or disappearance problem and accelerate the convergence speed.We set a sampling layer after the convolutional layer to reduce model parameters, prevent overfitting and increase the model receptive field. In the one-dimensional convolutional neural network model adopted in this study, the maximum pooling method was used. The main part of the output block is the Flatten layer and two fully connected layers stacked at the end of CNN as classifiers. The two fully connected layers use ReLU and Sigmoid as activation functions, respectively, and finally output the categories that predict the binary classification. The model used in this study uses the Dropout mechanism ([Krizhevsky et al., 2017](#_ENREF_6)) and the Batch Normalization (BN) method ([Ioffe and Szegedy, 2015](#_ENREF_4)) to enhance the stability and robustness of the model. BN is equivalent to Dropout in the overall effect of preventing overfitting (Ioffe and Szegedy, 2015[Ioffe and Szegedy, 2015](#_ENREF_4)). We added these regularization operations before the Flatten layer and before the last fully connected layer to improve the performance of the model. The L2 regularization penalty is also used in the full join layer to keep the weight values small and avoid overfitting.

**Training sample division method**

We recorded all the characteristic samples of drugs and their targets with potential therapeutic effects from the database as positive samples and assigned the label to 1. The construction of negative samples is to count the average number of targets n of all drugs by creating a non-binding drug-target relationship. For each drug, n randomly selected targets from which the targeting relationship was not determined were set to 1, and the rest were set to 0, while the substructure feature representation was kept unchanged, thereby constructing a negative sample set with a label of 0, so that the ratio of positive to negative samples was 1: 1. There may be unknown positive interactions between the selected negative samples, but the probability (1036 ÷ (223 × 470-1036) ≈ 0.010 %) is very less compared to the total unknown interactions in the dataset. Similarly, a data set with a positive and negative sample ratio of 1: 5 and 1: 10 was established by randomly selecting 5n and 10n positions to construct a random targeting relationship among all targets with undetermined targeting relationships. Finally, the positive and negative sets are combined to generate three training sets, each containing 446 samples（Table S5）.

**Performance test metrics formula**

$$\begin{aligned} \boldsymbol{ACC=}\frac{\boldsymbol{TP+TN}}{\boldsymbol{TP+TN+FN+FP}}\#\left( 1 \right) \end{aligned}$$

$$\begin{aligned} \boldsymbol{SEN=}\frac{\boldsymbol{TP}}{\boldsymbol{TP+FN}}\#\left( 2 \right) \end{aligned}$$

$$\begin{aligned} \boldsymbol{SPE=}\frac{\boldsymbol{TN}}{\boldsymbol{TN+FP}}\#\left( 3 \right) \end{aligned}$$

$$\begin{aligned} \boldsymbol{F}\boldsymbol{1=}\frac{\boldsymbol{2}\boldsymbol{TP}}{\boldsymbol{2}\boldsymbol{TP+FP+FN}}\#\left( 4 \right) \end{aligned}$$

$$\begin{aligned} \boldsymbol{PPV=}\frac{\boldsymbol{TP}}{\boldsymbol{TP+FP}}\#\left( 5 \right) \end{aligned}$$

$$\begin{aligned} \boldsymbol{NPV=}\frac{\boldsymbol{TN}}{\boldsymbol{TN+FN}}\#\left( 6 \right) \end{aligned}$$

$$\begin{aligned} \boldsymbol{MCC=}\frac{\boldsymbol{TP\cdot TN-FP\cdot FN}}{\sqrt{\left( \boldsymbol{TP+FP} \right)\boldsymbol{\cdot}\left( \boldsymbol{TP+FN} \right)\boldsymbol{\cdot}\left( \boldsymbol{TN+FP} \right)\boldsymbol{\cdot}\left( \boldsymbol{TN+FN} \right)}}\#\left( 7 \right) \end{aligned}$$

where, *TP*, *TN*, *FP*, and *FN* denote the number of [true positive](https://www.sciencedirect.com/topics/computer-science/true-positive), true negative, false positive, and false-negative samples, respectively.

**Reference:**

Cotto, K.C., Wagner, A.H., Feng, Y.Y., Kiwala, S., Coffman, A.C., Spies, G., Wollam, A., Spies, N.C., Griffith, O.L., Griffith, M., 2018. DGIdb 3.0: a redesign and expansion of the drug-gene interaction database. Nucleic Acids Res 46(D1), D1068-D1073.

Durant, J.L., Leland, B.A., Henry, D.R., Nourse, J.G., 2002. Reoptimization of MDL keys for use in drug discovery. Journal of chemical information and computer sciences 42(6), 1273-1280.

Gilson, M.K., Liu, T., Baitaluk, M., Nicola, G., Hwang, L., Chong, J., 2016. BindingDB in 2015: A public database for medicinal chemistry, computational chemistry and systems pharmacology. Nucleic acids research 44(D1), D1045-1053.

Ioffe, S., Szegedy, C., 2015. Batch Normalization: Accelerating Deep Network Training by Reducing Internal Covariate Shift. JMLR.org.

Kim, S., Chen, J., Cheng, T., Gindulyte, A., He, J., He, S., Li, Q., Shoemaker, B.A., Thiessen, P.A., Yu, B., Zaslavsky, L., Zhang, J., Bolton, E.E., 2021. PubChem in 2021: new data content and improved web interfaces. Nucleic acids research 49(D1), D1388-d1395.

Krizhevsky, A., Sutskever, I., Hinton, G.E., 2017. ImageNet classification with deep convolutional neural networks. Commun. ACM 60(6), 84–90.

Nair, V., Hinton, G.E., 2010. Rectified Linear Units Improve Restricted Boltzmann Machines. ICML.

Oughtred, R., Stark, C., Breitkreutz, B.J., Rust, J., Boucher, L., Chang, C., Kolas, N., O'Donnell, L., Leung, G., McAdam, R., Zhang, F., Dolma, S., Willems, A., Coulombe-Huntington, J., Chatr-Aryamontri, A., Dolinski, K., Tyers, M., 2019. The BioGRID interaction database: 2019 update. Nucleic Acids Res 47(D1), D529-D541.

Öztürk, H., Ozkirimli, E., Özgür, A., 2016. A comparative study of SMILES-based compound similarity functions for drug-target interaction prediction. BMC bioinformatics 17, 128.

Whirl-Carrillo, M., Huddart, R., 2021. An Evidence-Based Framework for Evaluating Pharmacogenomics Knowledge for Personalized Medicine. 110(3), 563-572.

Wishart, D.S., Feunang, Y.D., Guo, A.C., Lo, E.J., Marcu, A., Grant, J.R., Sajed, T., Johnson, D., Li, C., Sayeeda, Z., Assempour, N., Iynkkaran, I., Liu, Y., Maciejewski, A., Gale, N., Wilson, A., Chin, L., Cummings, R., Le, D., Pon, A., Knox, C., Wilson, M., 2018. DrugBank 5.0: a major update to the DrugBank database for 2018. Nucleic acids research 46(D1), D1074-d1082.

Zhou, Y., Zhang, Y., Lian, X., Li, F., Wang, C., Zhu, F., 2022. Therapeutic target database update 2022: facilitating drug discovery with enriched comparative data of targeted agents. 50(D1), D1398-d1407.
