# Supplementary material for: A deep learning drug screening framework for integrating local-global characteristics: A novel attempt for limited data
Source: Heliyon. 2024 Jul 14;10(14):e34244. doi: 10.1016/j.heliyon.2024.e34244 (PMC11315141; doi:10.1016/j.heliyon.2024.e34244)
Supplement: Multimedia component 2 [file mmc2.pdf]

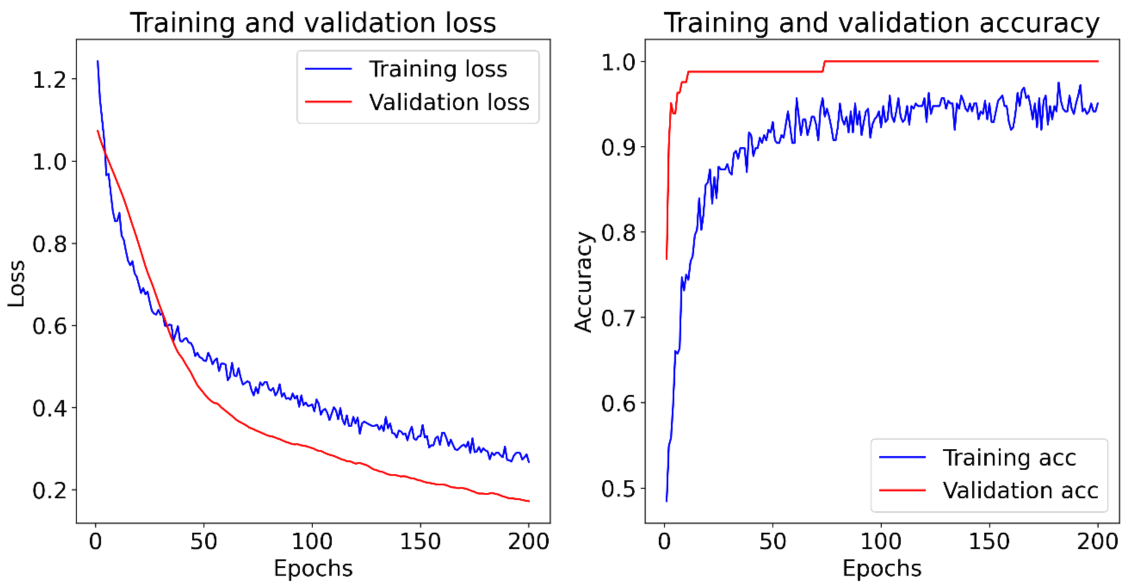

Figure S1.Loss values and accuracy change image of training set and test set

（Positive sample: negative sample =1:5）

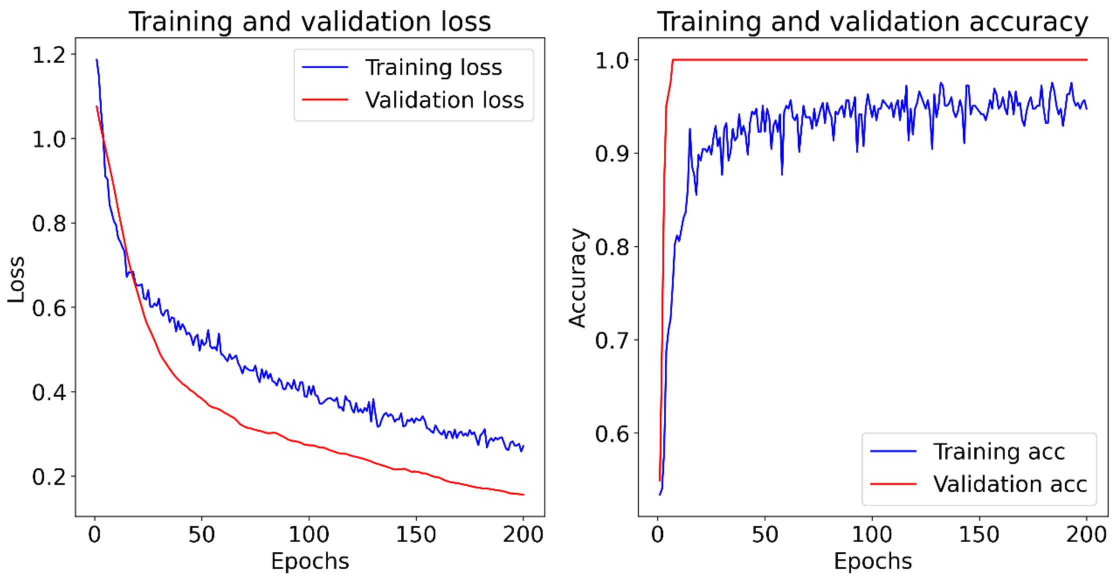

Figure S2. Loss values and accuracy change image of training set and test set

（Positive sample: negative sample =1:10）
